# Green synthesis and characterization of silver nanoparticles for reducing the damage to sperm parameters in diabetic compared to metformin

**DOI:** 10.1038/s41598-023-29412-3

**Published:** 2023-02-08

**Authors:** Iman A. Mohammed Ali, Ali Ben Ahmed, Hazim Ismail Al-Ahmed

**Affiliations:** 1 Laboratory of Applied Physic, Faculty of Sciences of Sfax, University of Sfax, 3018 Sfax, Tunisia; 2 Ministry of Higher Education & Scientific Research’s, Baghdad, Post Office PO Box 55509, Iraq; 3 Biotechnology Research Center, University of Al-Nahrain, Baghdad, 35095 Iraq

**Keywords:** Biotechnology, Plant sciences, Medical research, Materials science, Nanoscience and technology, Physics

## Abstract

The present study used physics to synthesize silver nanoparticles using aqueous extract of fresh garlic as reducing and as a stabilizing agent silver nitrate solution. This method has proven to be environmentally friendly and safe for the synthesis of stable silver nanoparticles. The acquisition of silver nanoparticles was confirmed by optical detection, that is, by changing the color of the liquid to transparent orange and then blackish brown. Then, the characterization was confirmed using other assays. In this study, it was found that the absorption peak of silver nanoparticles was at a wavelength of 420 nm and the particle size ranged between [50–350] nm. The surface roughness of silver oxide/silver nanoparticles was 9.32 nm with an average square roughness of 21.19 nm, and the energy dispersive spectra showed that the absorption peak was in the region of 3 keV, indicating that the nanoparticles contained crystalline silver. In this study, the stability of the silver nanoparticles was good, as ZP reached (− 19.5). The results confirm that the conductivity increases with the increase in frequency due to the high energy of the photons, which causes the electrons to vibrate in the energy levels and thus increase the energy in the mitochondria and increase the movement of sperm in the Diabetic mice treated with doses of silver nanoparticles. The toxic effect of silver nanoparticles has been evaluated in other studies, in addition to evaluating antioxidants, antifungals, treating cancer cells, regulating cholesterol levels, the effect of these nanoparticles on sex cells in pregnant female mice, heart tension, and many other tests. In this study, the activities and efficacy of silver nanoparticles on sperms were determined in male mice with diabetes caused by STZ, and the treatment period was long (35 days) so that the evaluation period was a complete life cycle of male sex cells and within a long period of time and at an average nano size. This has not been studied in other previous studies. The results indicate that the biosynthesis of silver nanoparticles using garlic plant led to positive results on sperm treatments by contributing to an increase in the number of sperm with reactivation and a decrease in abnormalities in addition to a decrease in mortality due to diabetes. This is evidence that the synthesis of silver nanoparticles using garlic plant size (50–350 nm) can treat impotence and be used in the future in the treatment of many diseases without side effects.

## Introduction

Nanotechnology is described in this study. It is a technique that has attracted a lot of interest in recent years. Nanoparticles represent all types of particles that fall within a diameter of 1–100 nm, and are made of carbon, metal, metal oxides, or organic compounds^1^. When compared with its larger counterpart (Bulk), nanoparticles have distinctive physical, chemical and biological characteristics due to their small size and large surface area, which led to improved interaction or stability during the physical and chemical process, increased mechanical strength and other factors^2–4^. Nanoparticles are distinguished by their different shapes and sizes^5^, in addition to their different diameters. Nanoparticles may be spherical or cylindrical in shape and can be tubular, spiral, flat or irregular in shape and size and range from 1 to 100 nanometers^6,7^. Nanotechnology is rapidly developing, and many intensive studies have been conducted to synthesize nanoparticles with distinctive characteristics in terms of cost, speed of completion and the distinctive characteristics of the resulting nanoparticles^8^. NPs have been used in a variety of applications including cooking utensils, renewable energies, agricultural pest control and have been used extensively in the medical field, treating a wide range of diseases, as well as transporting medicines and improving the quality of materials^9^. Silver nanoparticles are among the most important and most common nanoparticles due to the unique properties they possess, such as good conductivity and stability. They are also used in the manufacture of therapeutic alloys, in the treatment of burns and infections resulting from wounds, as anti-cancer cells, and against viruses, bacteria and free radicals^10^, and can be used in the manufacture of tools in contact with food, as a result, it can cause direct contact between silver nanoparticles and workers in this field, and this in turn can cause semi-chronic toxic effects and may interact with the health of the organism, as it was found that AgNPs have the ability to precipitate in The kidneys, testicles, lungs, heart and other organs of the body result in the generation of reactive oxygen species in living cells and can cause immunological or neurotoxicity^11^, on the other hand, many studies have shown that exposure to silver nanoparticles can treat many diseases resulting from External and chronic causes, such as diabetes, pressure, or immune diseases^12–14^. There are few studies that show the effect of silver nanoparticles on sex cells, as many have shown Among the studies, there is a relationship between the amount of the dose once and the approved period in which the infected body is exposed to a quantity of therapeutic nanoparticles again and between the health of the sperm^15^. There are various methods for the synthesis of nanoparticles, including chemical methods, in which highly dangerous compounds are used and toxic for the purpose of minimization and stabilization, causing environmental damage in addition to being expensive and time consuming with high energies. Biological and physical methods are among the ideal methods used for the synthesis of nanoparticles because they are simple, harmless, environmentally friendly, and effective at the same time^16,17^. Research and studies have increased to use the biophysical method to manufacture nanoparticles, as it is considered the best to use effective plant-derived compounds such as polyphenols, flavonoids, anthocyanins, ellagic acid and other plant materials that can improve the properties of silver nanoparticles and reduce toxicity resulting from the use of nanoparticles. Metals and salts used in the manufacture of commercial silver nanoparticles^18^.

Our current study focuses on the synthesis of silver nanocomposites derived from biological sources to reduce harmful ions and toxicity resulting from substances that are a source of stabilization and reduction^19^. An aqueous extract of garlic was used^20,21^, and testing the toxicity of silver nanoparticles on the sperm of healthy mature mice, and evaluating the role of therapeutic silver nanoparticles in stimulating sperm movement and reducing deformities in sperms as a result of STZ-induced diabetes, and possibly death in some of them and a decrease in the number of sex cells. Treatment with manufactured silver nanoparticles was tested with a long treatment period of up to 35 consecutive days and at a rate of one dose per day, that is, according to the entire life cycle of the sperm, which was not observed in another research. The results indicate that there is no significant toxicity resulting from the silver nanoparticles, and this is considered a success in using a quick and inexpensive method of synthesis, it can be used in the treatment of many diseases and negative effects resulting from diabetes.

## Experimental section

### Synthesis materials

Silver nitrate, Streptozotocin obtained from Sigma Corporation, USA industry was used. Fresh garlic was purchased from the traditional medicine store in Baghdad. The garlic plant sample was classified by an expert at the University of Baghdad, College of Science, Department of Biology (AlliumL type/Alliaceae family)^22^. Ethanol was taken from DUKSAN Company The deionized water was used during the preparation of the aqueous extract and all the tools used were washed using distilled water and left to dry using a hot oven before use.

#### Prepare fresh garlic leaf extract

Fresh garlic was used after washing several times with deionized water (DIW) to remove dust particles, then the plant was left in the air to dry to remove residual moisture. Dry garlic was ground using an ordinary grinder. 15 g of finely ground garlic powder was dispersed in 500 ml DIW, which is of very high purity, using a magnetic stirrer for 30 min at 100 °C. Then, the solution was filtered through filter paper and centrifuged at 4000 rpm for 30 min to remove any impurities and obtain a clear solution. The extract is kept in the refrigerator for later use in the preparation of silver nanoparticles^23^.

#### Synthesis of silver nanoparticles by green method

The preparation was carried out according to^24,25^ with some modification. 2 g of silver nitrate was dissolved in 25 ml of distilled water by a 600 RPM magnetic stirrer for 30 min. After that, we add 25 ml of garlic extract gradually with continuous magnetic stirring for an hour at 80 °C. Then a precipitate will form and the color of the solution will turn black. The solution was then left overnight and the precipitate was separated by centrifuge and washed with water and ethanol more than once. The precipitate is dried in an oven at 85 °C for 4 h.

### Characterization techniques

#### X-ray diffraction analysis (XRD)

It is a technique used to study the arrangement of atoms inside crystals, where X-ray diffraction of silver nanoparticles was measured using XRD analysis. The examination was carried out by placing the sample in a centrifuge at 10,000 rpm for 15 min. The precipitate was collected and the resulting silver pellets dried at 50 °C in an oven. The size of silver nanoparticles is calculated using the Scherer equation, which is shown below, $D = K.\lambda/\beta.{\text{cos}}\theta$, knowing that the constant k (geometric factor) is equal to $0.94$^24^.

#### Field emission scanning electron microscope (FE-SEM)

The structure of silver nanoparticles and the size and shape of the nanoparticles were studied using scanning electron microscope (EDS-Mapping-Line-EBSD) made in Germany. The examination was carried out after placing the sample in a centrifuge at a rotation speed of 10,000 rpm for 15 min, after which the sample was washed with distilled water and dried at a temperature of 50 degrees Celsius. The sample was placed on a platinum mesh coated with palladium and the sample was analyzed by the radiation passing through the sample and the image was at a dispersion spectrum of (250 INCA Energy)^26^.

#### Atomic force microscopy (AFM)

Atomic force microscopy was used to examine the surface morphology of silver nanoparticles produced by garlic extract. The examination was carried out after the examined sample was dispersed and placed on a small glass slide under the microscope and at room temperature^27^.

#### Transmission electron microscopy (TEM)

It is a very powerful technique in materials science that can be described by a beam of high-energy electrons passing through very thin samples. The properties of silver nanoparticles in terms of particle shape and size were studied using TEM technology. An amount of the dried precipitate of nano silver was dissolved in ethanol alcohol, the suspension was placed in the ultrasonic bath for 15 min, then a drop is taken from the suspension and placed on a carbon-coated copper grid. We note that after the sample dries and forms a partially transparent layer, the sample is examined and the resulting image is formed from the shadow of the electron beam falling on the sample^28^.

#### UV–visible spectra analysis

The UV–visible spectrum of stable silver nanoparticles and aqueous extract of fresh garlic plant was recorded using a UV–Vis spectrometer (Shimadzu-Japanese uv-2450) at 1 nm resolution to ensure the reduction of silver Ag + ions to AgO using garlic extracts as a reducing agent. Samples were scanned in the 300–800 nm range, with a scanning speed of 475 nm/min, at 1 cm optical path and at room temperature. UV–vis absorption spectra were recorded 24 h after incubating the AgNO_3_ solution with garlic extract. Distilled water was used as a blank reference for background yellowing from other sources^29^.

#### Zeta potential measurement

The surface electric charge of Ag NPs was determined by measuring the most stable particles when electrostatic repulsion occurs between the particles. Zeta potential was determined using HAS 300. Zeta sizer based on photon correlation spectroscopy^30^. The analysis time was 60 s, and the average zeta potential was determined. Dispersion was determined as such without dilution.

#### Dynamic light scattering (DLS)

The hydrodynamic diameter of silver nanoparticles in solution was determined by dynamic light scattering (DLS) and multiple scattering laser diffraction method. Malvern Zeta sizer from Origin/Germany was used^31^.

### Biotechnological part

#### Animals experiment

Thirty healthy male albino mice weighing about 30 ± 5 g used in the experiment were purchased from Biotechnology Research Center/Al-Nahrain University. All animals were kept in standard conditions of 22 ± 3 °C with a constant 12 h dark and 12 h light exposure cycle, and in a controlled environment at an equilibrium humidity of 50 ± 5%. The animals were left for a week to acclimatize to the experimental conditions while being provided with the standard healthy diet of food and water. The experiment was conducted in the animal house of Al-Nahrain University.

#### Stimulating experimental diabetes mellitus in albino mice

Diabetes was induced in all male experimental albino mice, except for the healthy control, by giving them a single dose of fresh streptozotocin after being dissolved in saline in an amount $\left( {200 \;{\text{mg}}/{\text{kg}}} \right)$ of body weight. This dose stimulated diabetes mellitus in male rats, in which hyperglycemia was measured by measuring blood glucose 3–6 days after streptozotocin administration. Using a glucose meter, blood was drawn from the tail vein of mice White mice that showed a blood glucose level higher than 245 mg/dL were taken and with this methodology these animals were selected alone for the current study^32^.

#### Sperm analysis

The mice were divided into sex groups that were forced to observe the mice. The first group is the negative control group shown, watching these mice. The second group, control diabetic mice, took doses of streptozotocin, the third group was diabetic and treated with metformin (600 mg/kg body weight/day) for 28 days, the fourth group was diabetic and treated with Silver and the fifth was diabetic and treated silver nanoparticles (2 mg/kg body weight/day)^33^. Sperm motility, normal and damaged morphology, and sperm count were examined, and the number was found to be at least 150 sperms from each mouse. Sperm activity and motility analysis was performed by Makler Chamber and using a light microscope (Olympus Corporation, Tokyo, Japan). Where motility was expressed as fast (Grade A) and slow (Grade B) sperm, non-progressive (Grade C) and non-motile (Grade D) sperm. On the other hand, the shapes of sperms were identified by adjusting the optical microscope to magnification × 40^34^.

### Ethical declaration

The authors declare that: (1) All methods were carried out in accordance with relevant guidelines and regulations. All experimental protocols were approved by ethics committee in Biotechnology research center, at University of Al-Nahrain, Baghdad 35,095 Iraq: Application number 60, Reference number 21-3, date 20 May 2022. (2) All methods are reported in accordance with ARRIVE guidelines for the reporting of animal experiments. (See attached file: “Research Ethics Checklist (Animals)”).

## Results and discussion

### XRD analysis

**Figure 1:**
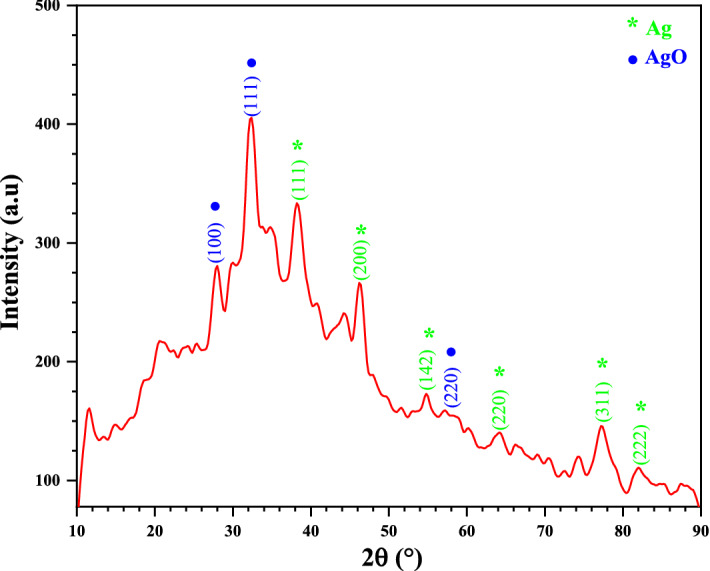
XRD pattern of Ag/AgO NPs.

X-ray diffraction is an important technique for determining crystal structure. It is used to determine the atomic arrangement, lattice parameters, crystal size^35^. Figure 1 shows the pattern of Ag/AgO NPs prepared by the biosynthesis method. There are (8) peaks are shown with different intensities. The diffraction angles at 27.9°, 32.2°, and 54.72° corresponding to planes (100), (111), and (220) respectively, confirm the behavior of AgO Nps and agreement with JCPDS (01–076-1489), while, the diffraction angles at 38.2° and 46.3°, 34.74°, 77.23°, and 81.82° corresponding to planes (111), (200), (220), (311), and (222) respectively. These results illustrated the AgNPs has been prepared, and agreement with the JCPDS (00-001-1167). The crystallite size of Ag/AgO NPs was calculated from the full width half-maximum, Bragg reflections by the Debye–Scherrer equation^36^:

1 $$D = \frac{0.9\lambda }{{\beta cos\theta }}$$

where $D$ is the crystallite size, $\lambda = 1.5406$ Å is the wavelength of X-ray, $\beta$ is the full width half maximum (FWHM) of the peak in radians, and $\theta$ is the Bragg angle. The crystallite size of Ag/AgO NPs are shown in Table 1.

**Table 1:** The crystallite size of Ag/AgO NPs.

Sample	$2\theta \left(^\circ \right)$	(hkl)	FWHM (°)	$D \left( {{\text{nm}}} \right)$
Ag/AgO* NPs	27.96	(100)*	0.590	13.88
	32.28	(111)*	0.590	14.02
	38.20	(111)	0.590	14.25
	46.31	(200)	0.590	14.65
	54.72	(220)*	0.095	94.19
	34.74	(220)	0.095	87.65
	77.23	(311)	0.092	110.55
	81.82	(222)	0.090	116.84
$D_{average} \left( {{\text{nm}}} \right)$				30.20

### FE-SEM–EDS analysis

**Figure 2:**
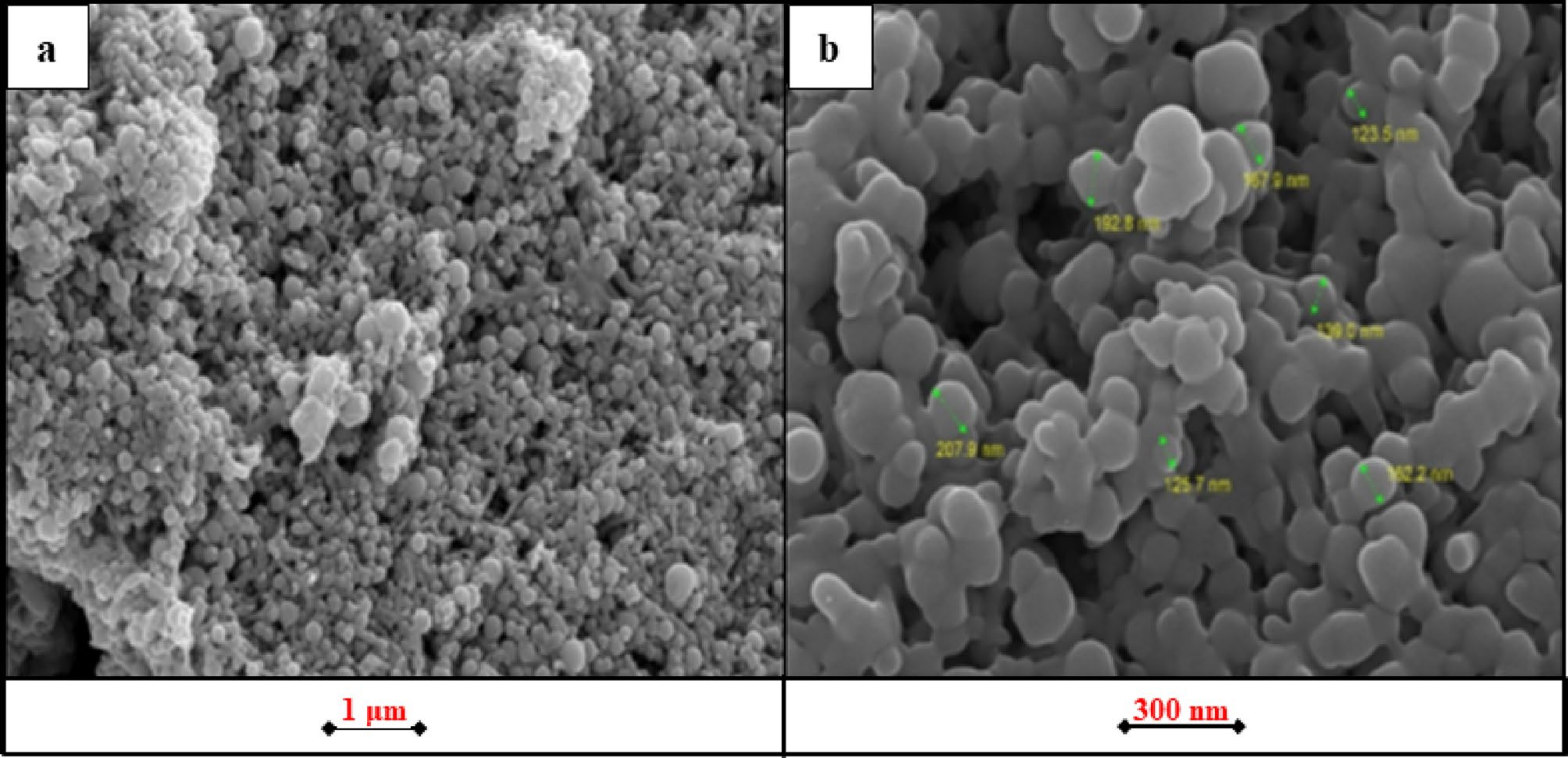
FESEM images of AgNPs at (**a**) 1 µm and (**b**) 300 nm.

The field emission scanning electron microscope (FESESM) provides the ability to study the topography of the surfaces of nanomaterials and determine the possibility of their application in different fields. The morphological images of Ag/AgO NPs created using the biosynthetic process are shown in Fig. 2a (1 μm) and Fig. 2b (100 nm). The findings indicate the existence of spherical nanoparticles, which resemble clusters of spherical nanoparticles, with different nano-diameter.

**Figure 3:**
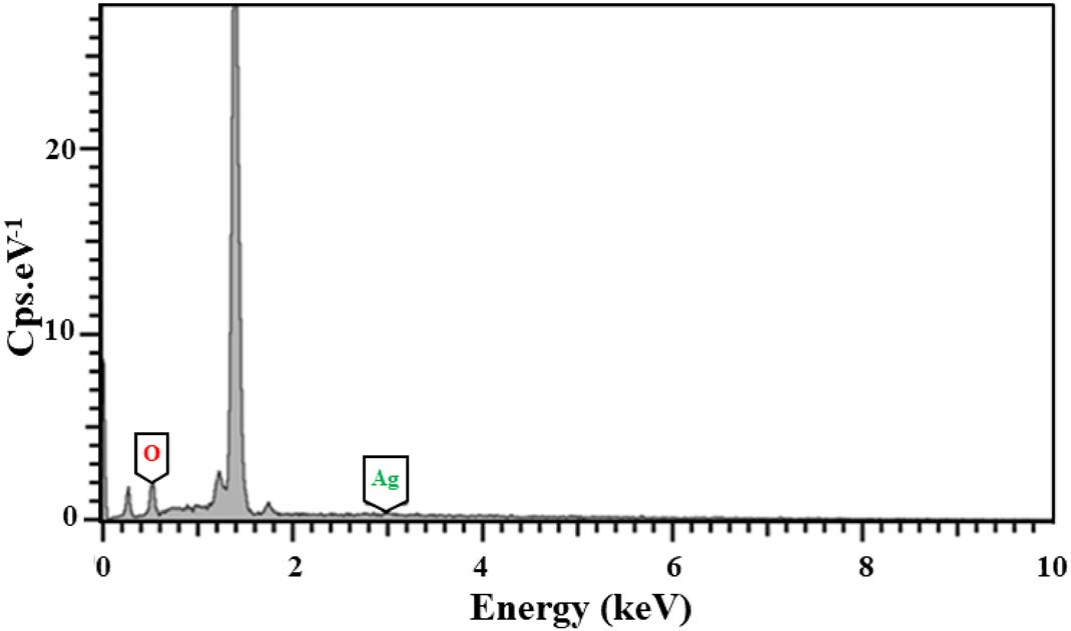
EDS of Ag/AgO NPs.

AgNPs existence and crystalline structure were study by using EDS analysis. It is widely known that surface Plasmon resonance causes Ag spherical nanoparticles to have a characteristic optical absorption peak about $3{ }\;{\text{keV}}$. Figure 3 displayed the absorption peak in the $3{ }\;{\text{keV}}$ area, demonstrating that NPs were made of crystalline silver.

The presence of a high content of oxygen may be attributed to the presence of silver oxides in the prepared sample, these results are consistent with the results of XRD analysis. The present results are consistent with the results of the work^37^.

### TEM analysis

**Figure 4:**
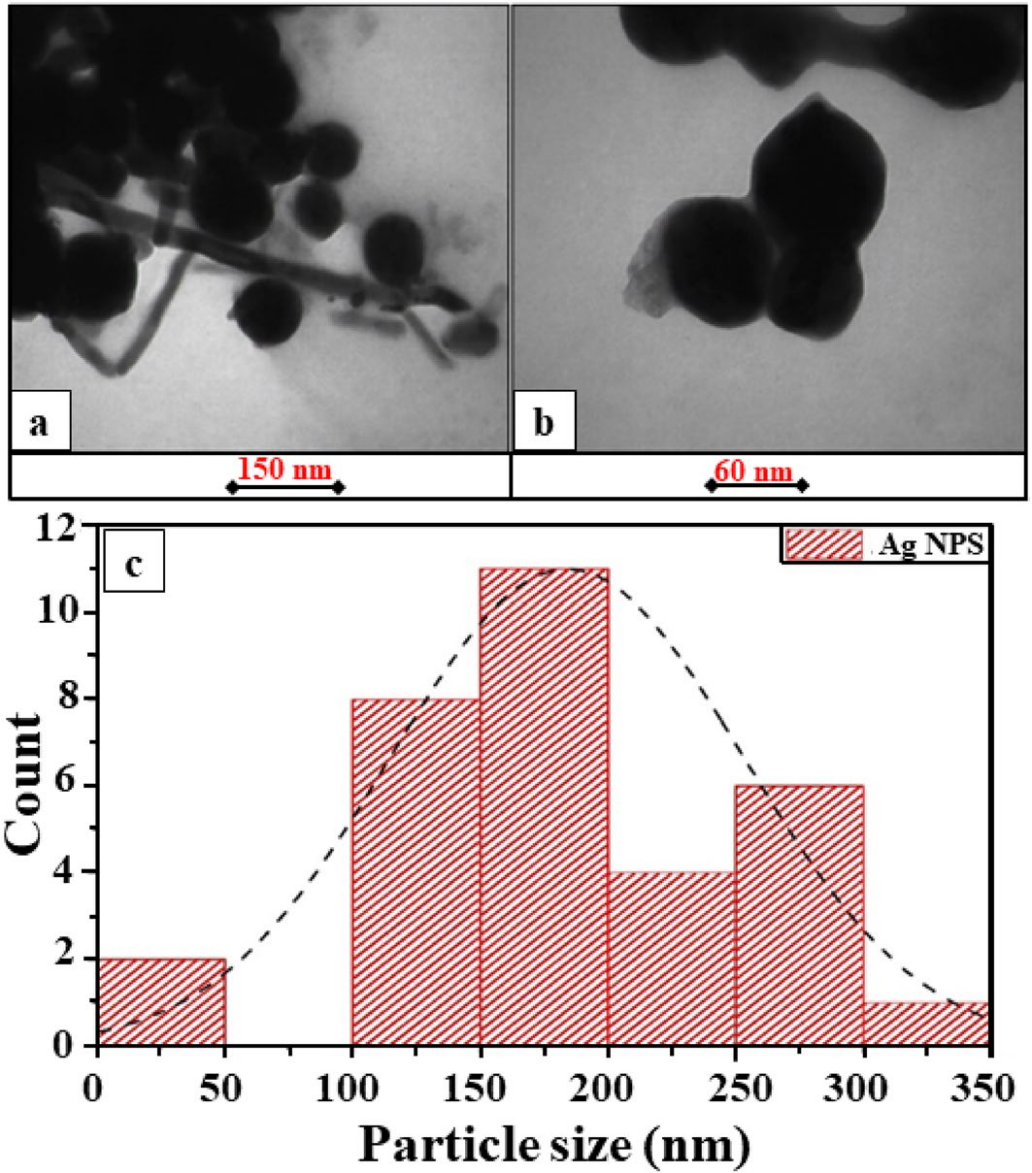
TEM Images of (**a**,**b**) Ag/AgO NPs prepared by biosynthesis method with different magnification, and (**c**) the histogram size distribution.

TEM is one of the advanced analytical measurement tools used for imaging and distinguishing the size and shape of nanoparticles^38^. Figure 4a,b show the TEM Image of Ag/AgO NPs prepared by the biosynthesis method. The results show that the nanoparticles have a spherical shape and with some aggregation. Figure 4c explains the histogram size distribution on Ag/AgO NPs with particles size ranging from $\left[ {50 - 350 } \right] \;{\text{nm}}$.

### AFM analysis

**Figure 5:**
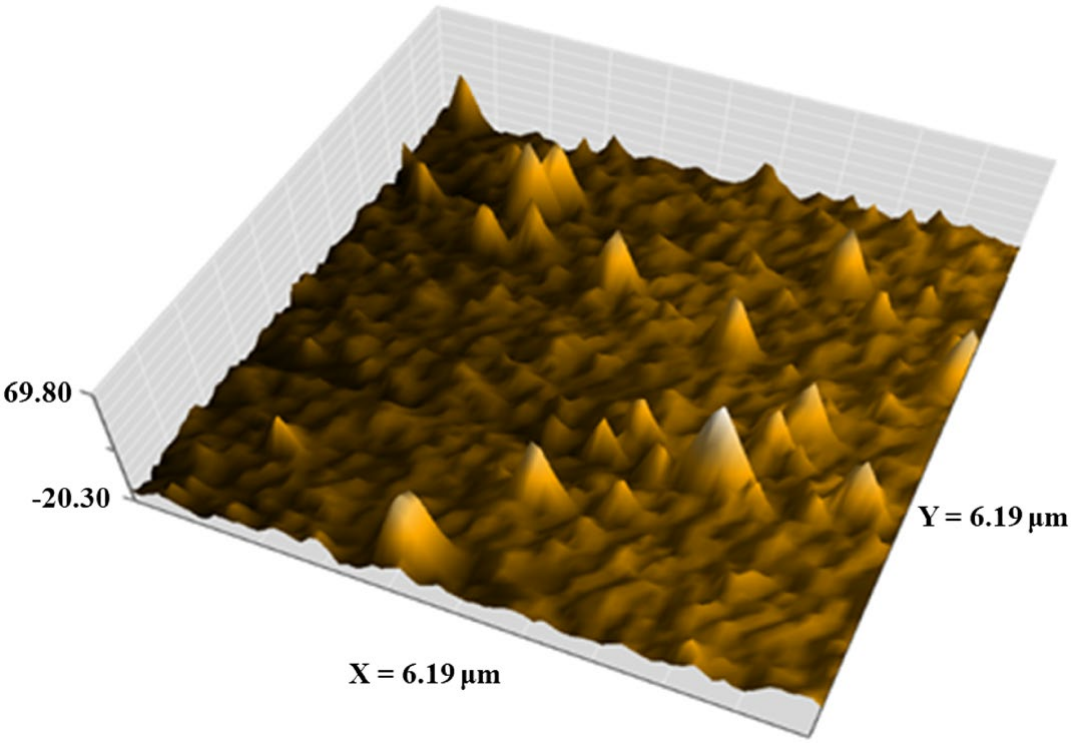
AFM images of AgO/AgNPs.

The roughness and surface morphology of AgO/AgNPs are indicated in the AFM images, and Fig. 5. This image was carried out by Naio Nanosurf software, version 3.10.0.28^39^. The results showed that the surface roughness of AgO/AgNPs was $9.32\;{\text{nm}}$ with an average square roughness of $21.19\;{\text{nm}}$, as shown in the Fig. 5. The present results are consistent with those of the TEM analysis.

### Optical properties

#### UV–Visible spectra analysis

**Figure 6:**
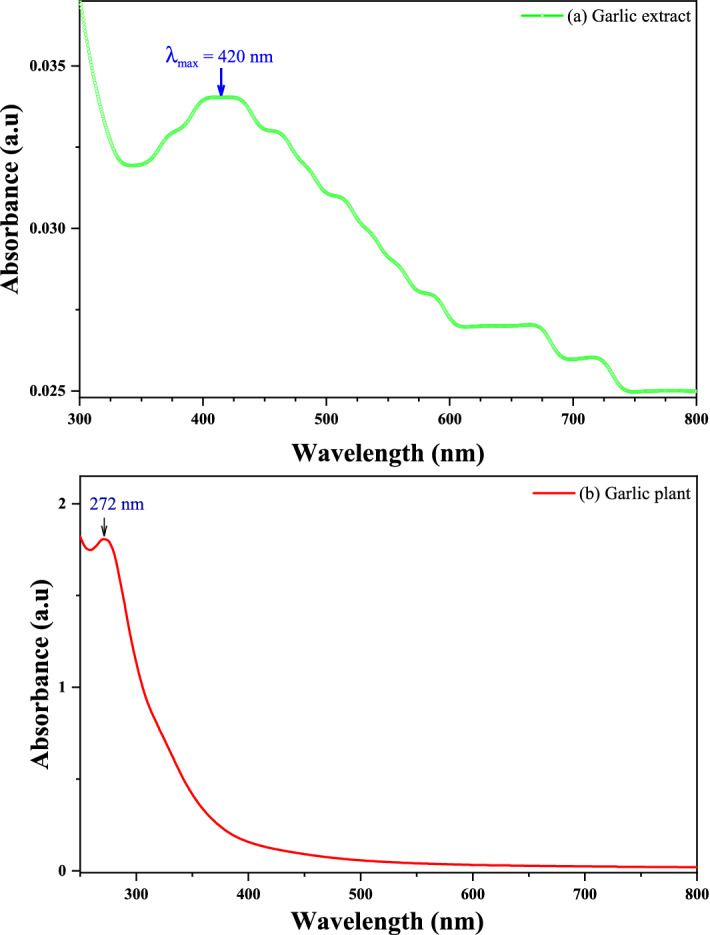
UV–visible absorbance spectra of AgO/AgNPs (**a**) extract garlic and (**b**) garlic plant.

Figure 6a shows the optical absorption spectrum of AgO/AgNPs prepared using garlic extract. The results show that there is an absorption peak at the wavelength $420\;{\text{nm}}$ that can be attributed to the presence of the surface plasmon resonance (it is the result of the collective movement of free electrons in the silver when light falls on it), which is a characteristic of AgNPs This result is close to the source^40^. The present results are consistent with the results of the work^41,42^. Figure 6b shows the absorption spectrum at a wavelength range (250–800) nm of garlic plant. The peak of the optical absorption spectrum was observed at wavelength 272 nm when using an aqueous garlic plant extract, and this is close to the studies presented in^43^.

#### Optical absorption coefficient

The attenuation of light intensity as it passes through a substance is described by the absorption coefficient $\left( \alpha \right)$. It may be thought of as the total of a material's absorption cross-sections for an optical process per unit volume. The optical absorption coefficient $\left( \alpha \right)$ can be calculated by the following equation^26^.

2 $$\alpha \left( \lambda \right) = \frac{A}{d} \times ln\left( {10} \right)$$

where $A$ is absorbance, $d$ is thickness and $ln\left( {10} \right) = 2.30$.

**Figure 7:**
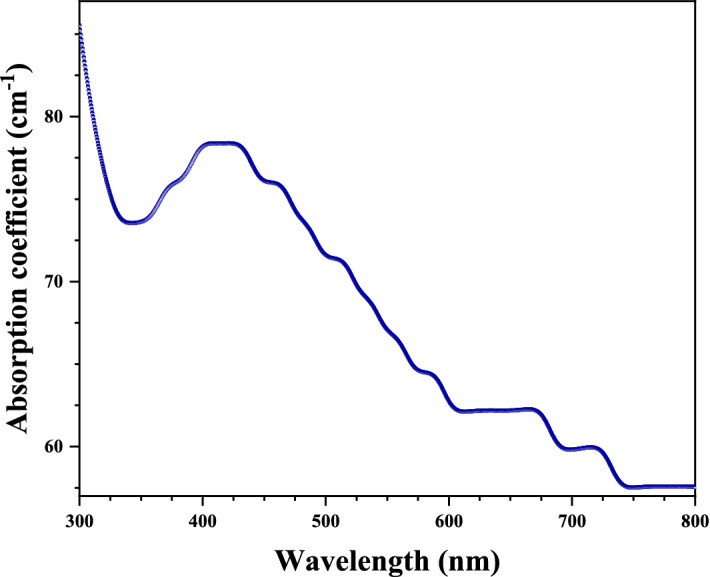
Variations of absorption coefficient with wavelength of AgO/AgNPs.

Figure 7 shows the relation between the optical absorption coefficient $\left( \alpha \right)$ and the wavelength of AgO/AgNPs prepared by garlic plant extract. The results indicate a peak absorption coefficient at the wavelength of $420\;{\text{nm}}$ with a high absorption edge close in the ultraviolet region. This curve represents the optical absorption coefficient (α) behavior of AgNPs^44,45^.

#### Urbach energy

**Figure 8:**
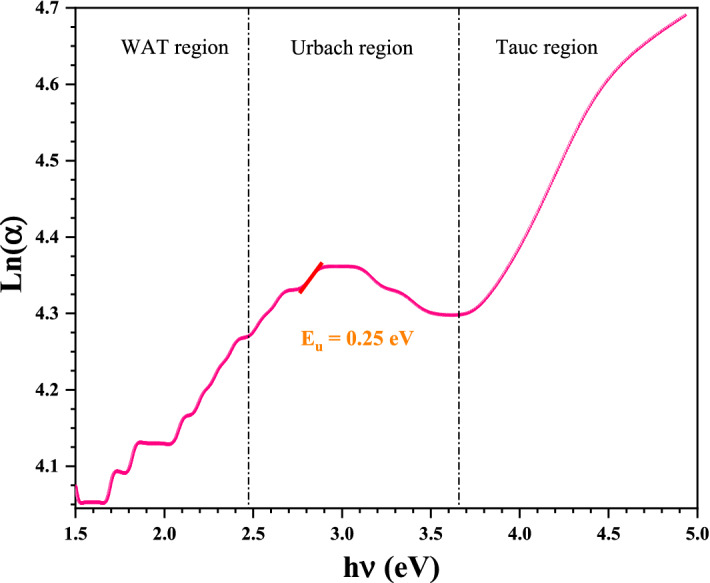
Variations of Ln(α) with incident photon energy of AgO/AgNPs.

Figure 8 shows the $Ln\left( \alpha \right)$ versus $h\nu$ plots obtained for the Ag nanoparticles thin film sample. This plot can be divided into three regions for analysis. The first region belongs to the weak absorption (WAT-Region). It represents the transitions that take place from a tail state located above the valence band to another tail state located below the conduction band, and/or from a tail state located below the conduction band to another tail state located above the valence band. In this WAT region, $\alpha$ follows $h\nu$ according to the following relationship^46^:

3 $$\upalpha \left( {{\text{h}}\upnu } \right) = \upalpha _{0} {\text{e}}^{{\left( {\frac{{{\text{h}}\upnu }}{{{\text{E}}_{{{\text{WAT}}}} }}} \right)}}$$

where $\alpha_{0}$ is a constant, $h\nu$ is the photon energy and $E_{WAT}$ represents the weak absorption tail energy.

The Urbach region (U region) (Fig. 8) represents the electronic transitions that take place from an extended valence band state to another tail state below the conduction band and/or d from a conduction band state extended to another tail state above the valence band. The $E_{u}$ can be calculated by the following equation^46^:

4 $$\upalpha \left( {{\text{h}}\upnu } \right) = \upalpha _{0} {\text{e}}^{{\left( {\frac{{{\text{h}}\upnu }}{{{\text{E}}_{{\text{u}}} }}} \right)}}$$

where $\alpha_{0}$ is a constant, $h\nu$ is the photon energy and $E_{u}$ is the Urbach energy.

5 $${\text{Ln}}\left( \upalpha \right) = \frac{1}{{{\text{E}}_{{\text{u}}} }}{\text{h}}\upnu + {\text{Ln}}(\upalpha _{0} )$$

The Urbach energy was calculated by the inverse of slope to the curve. In Fig. 8 we have the Urbach energy $\left( {E_{u} = 0.25\;{\text{eV}}} \right)$ this low value indicates low density of localized states in the bandgap of Ag nanoparticles thin film. So minimal impurities in our prepared Ag/AgO film.

#### Optical bandgap analysis

The determination of the optical bandgap $E_{g}$ was based on this Tauc formula:

6 $$\left( {\alpha h\nu } \right)^{2} = \beta \left( {h\nu - E_{g} } \right)$$

**Figure 9:**
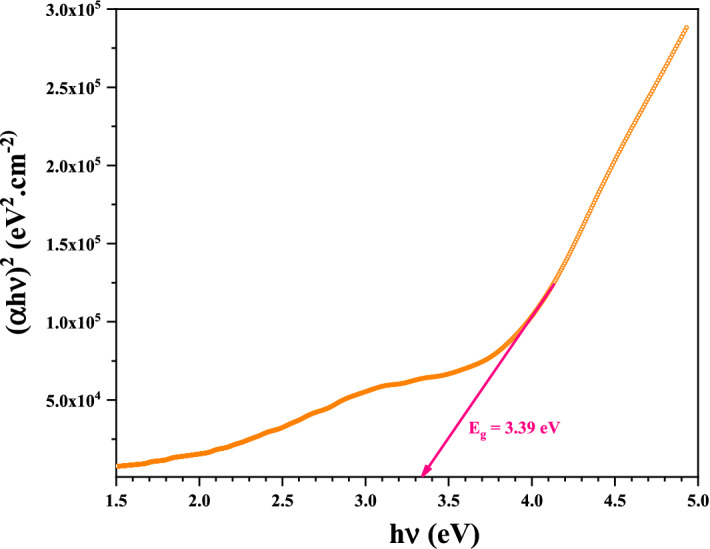
Tauc plot of AgO/Ag NPs prepared by garlic plant extract.

After plotting $\left( {\alpha h\nu } \right)^{2}$ in function of the photon energy $\left( {h\nu } \right)$, the bandgap value could be determined using the extrapolating of the linear portion to $\alpha = 0$. As can be seen in Fig. 9, the Tauc plot obtained for the Ag nanoparticles thin film sample. It was obtained from the T region in Fig. 8 as it represents the longest transition. Figure 9 show the relation between the photon energy (eV) and $\left( {\alpha h\nu } \right)^{2}$ of AgO/AgNPs, by drawing the tangent with the x-axis the direct optical energy gap can be calculated. From the Fig. 9, the results confirm that the energy gap directly was $3.39\;{\text{eV}}$, this meaning the sample needs less energy to stimulate the electrons to move between the energy bands. The current results are in agreement with the results of work^47^.

#### Refractive index

It has been reported that, the refractive index $n_{r}$ is a fundamental parameter of optical materials that plays a very important role in optical device designing. Thus, controlling the refractive index of optical nanomaterials makes them convenient for a wide range of applications in industrial and medical applications such as display devices, light-emitting diodes (OLEDs), optical communications, and antibacterial activities. The band gap values can be used to calculate a high frequency refractive index $n_{r}$ according to the empirical relation applicable to different varieties of compounds:

7 $$n_{r} = \frac{1}{{T_{s} }}{ } + \sqrt {\frac{1}{{T_{s} - 1}}} = \frac{1}{{10^{ - A} \times 100}} + { }\sqrt {\frac{1}{{10^{ - A} \times 100 - 1}}}$$

where $T_{s}$ is the percent transmittance and A is absorbance.

**Figure 10:**
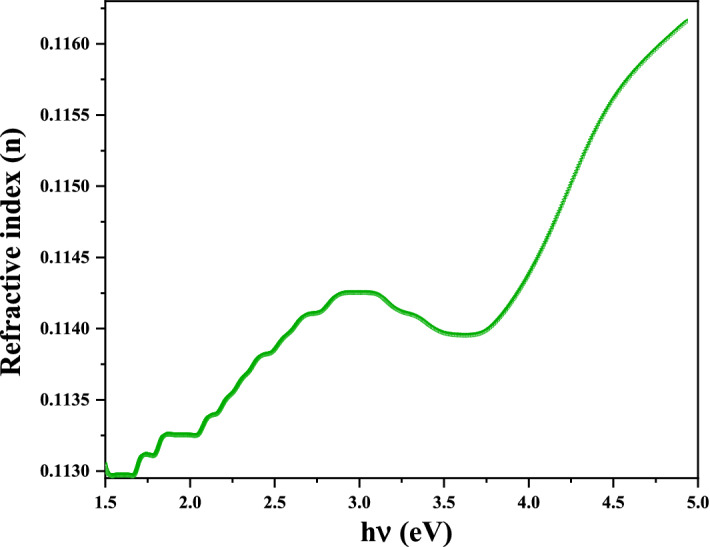
Plot of refractive index as function energy of AgO/Ag NPs.

As can be seen Fig. 10, the value of the refractive index for the Ag-NPs in the range $0.113 - 0.116$. This is close to the usual value of silver $\left( {0.135} \right)$ ^48^. The difference is that our compound is formed by AgO nanoparticles in addition to Ag nanoparticles.

#### Optical conductivity

The optical conductivity $\left( {\sigma_{opt} } \right)$ for this sample was calculated using the absorption coefficient $\alpha$, and the refractive index $n$ data using the following relation^49^:

8 $$\sigma_{opt} = \frac{{\alpha n_{r} c}}{4\pi }$$

where $c$ is the velocity of light in free space, $\alpha$ is the absorption coefficient and ${ }n_{r}$ is the refractive index.

**Figure 11:**
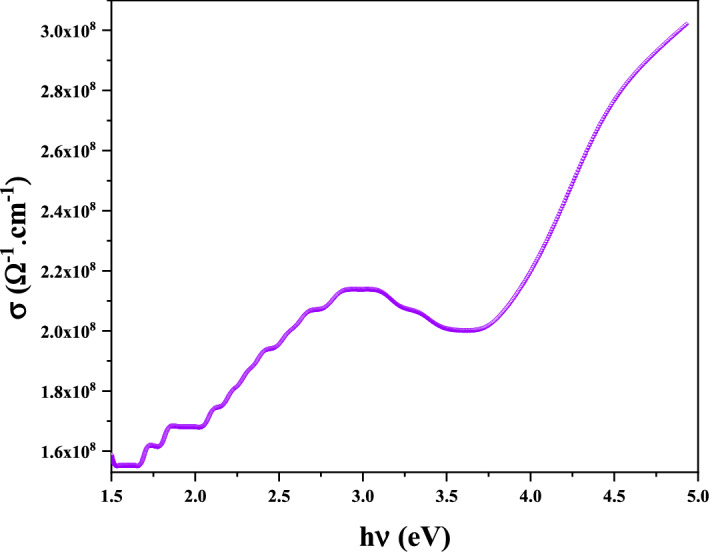
The relation between optical conductivity and energy of AgO/Ag NPs.

The optical conductivity of a material determines the relationship between the amplitude of the induced electric field and the density of the induced current in the material for any given frequency. This linear response function is a generalization of electrical conductivity, which is often considered in terms of static electric fields with slow or time-independent differences. It has to do with how conductive a material is, or how much electricity can flow through it. The conductivity of a particular material depends on the frequency of the electric field (that is, how fast it changes)^50,51^. Figure 11 shows the photoconductivity as a function of the photon energy (frequency) of silver nanoparticles.

The results confirm that the conductivity increases with increasing frequency due to the high energy of the photons, causing the electrons to vibrate in the energy levels.

### Zeta potential measurement

**Figure 12:**
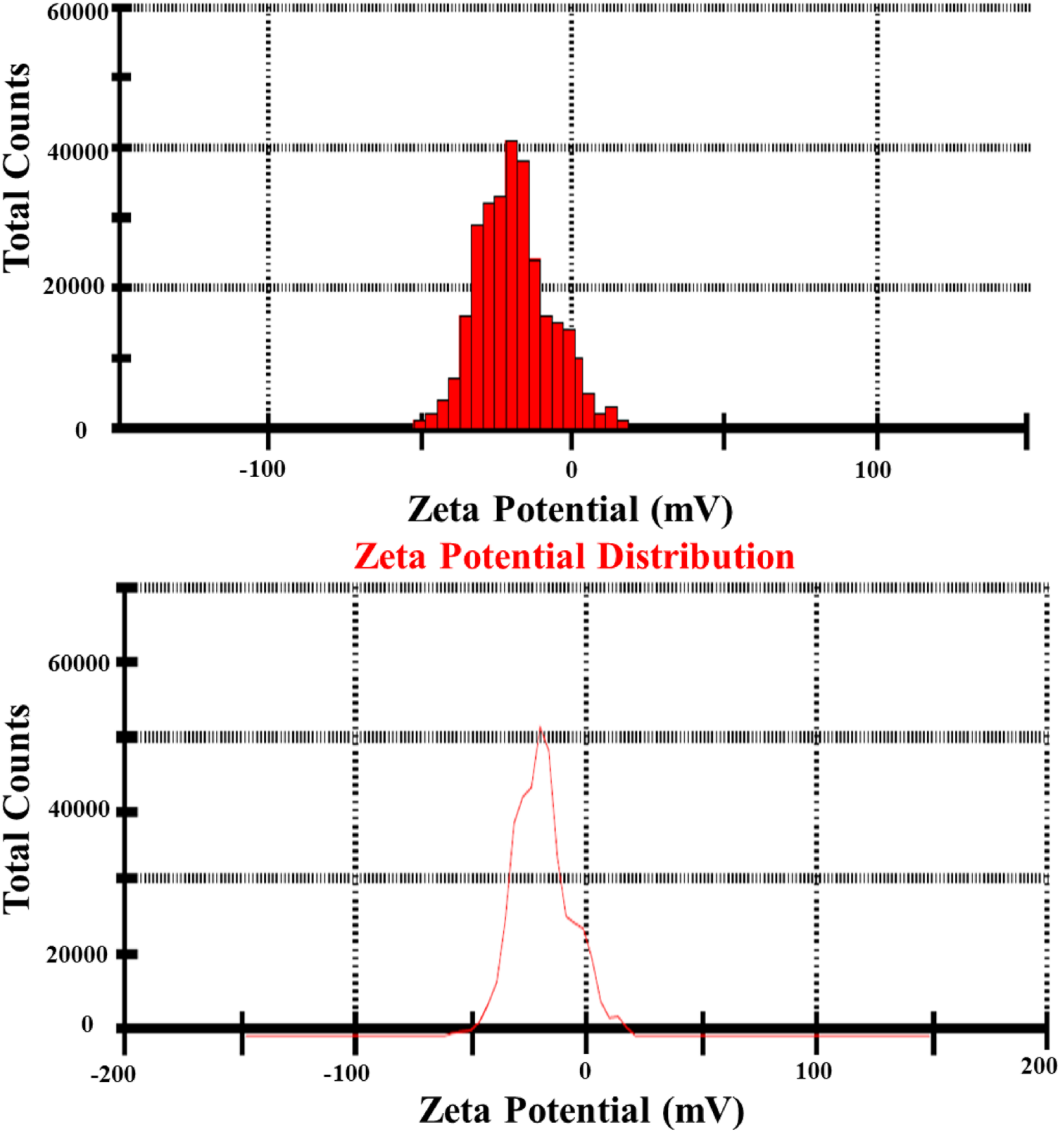
Zeta potential values of (A) AgO/AgNPs.

Figure 12 shows the surface charge measurements of both AgO/AgNPs. The results confirm the dispersion of AgO/AgNPs, which have a zeta potential of − 19.5 (mV) and a mobility of − 1.532 (μmcm/Vs). The present results show a good dispersion state of NPs in liquids^52^. The negative value in this test confirms the occurrence of repulsion between the silver nanoparticles and proves that they are very stable.

### Dynamic light scattering (DLS) analysis

**Figure 13:**
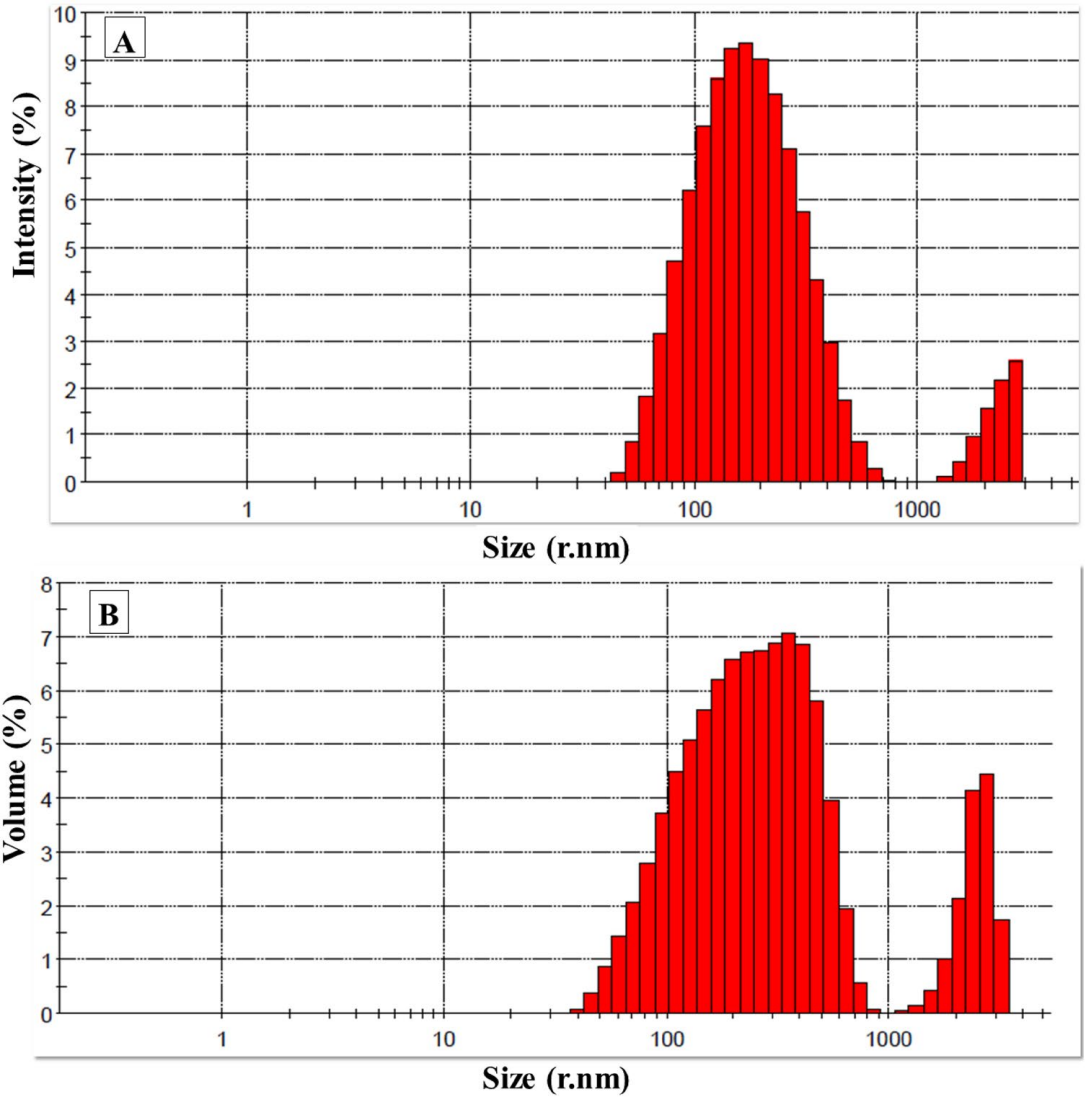
Size Distribution of Ag/AgONPs by (**A**) Intensity and (**B**) Volume. At Temperature 25 °C and Duration 60 s.

Figure 13 shows the size distribution (by intensity and volume) of AgO/AgNPs prepared by the plant extract. From the figure, the results confirm that the highest peaks of AgO/AgNPs distribution were between 95 and 310 nm. The present results are in close agreement with the results of TEM analysis.

### Sperms parameters

**Figure 14:**
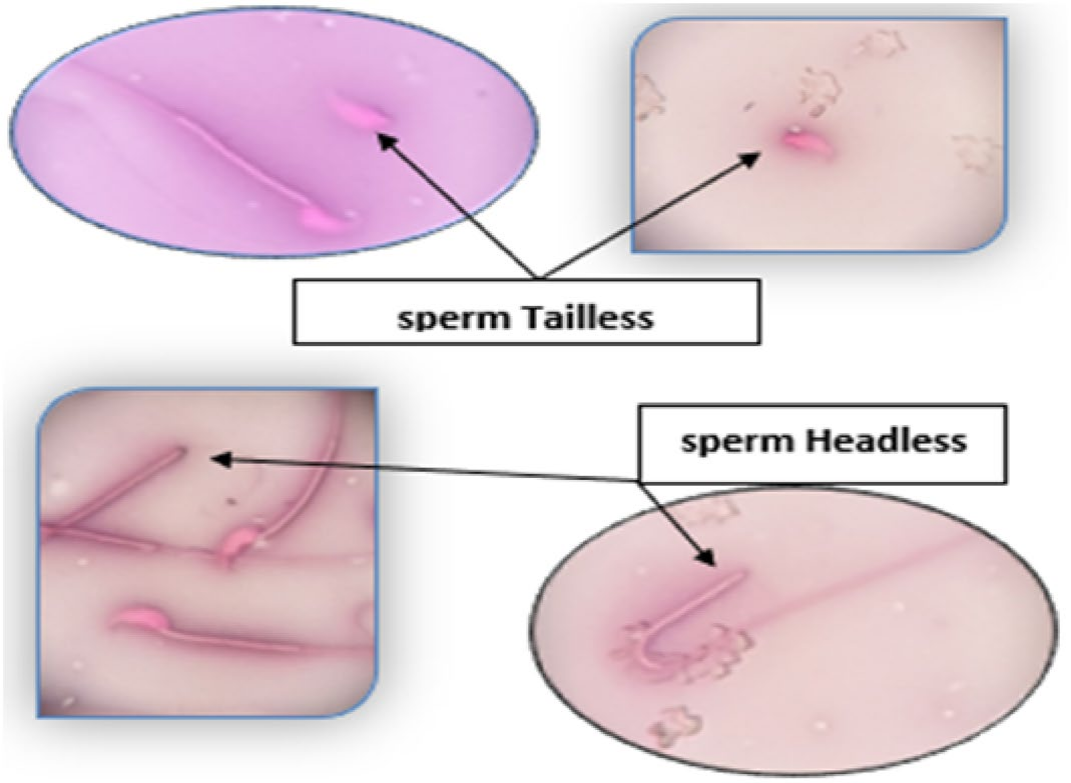
Sperm without tail & sperm without Head. Slide stained with Nigrosine and Eosin stain (100×).

**Figure 15:**
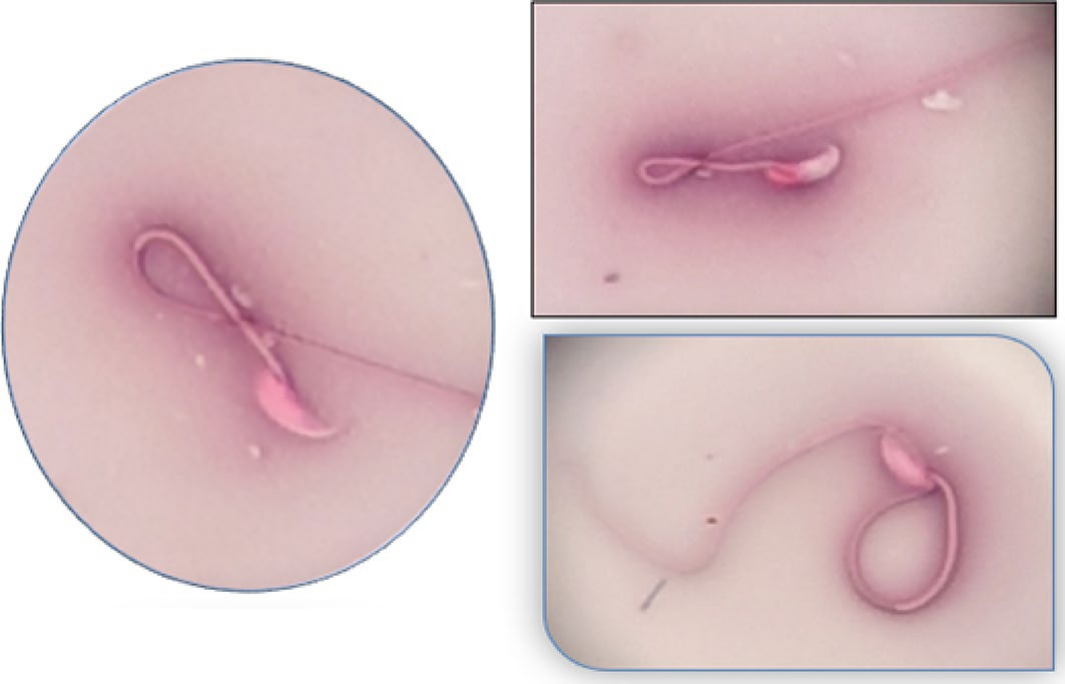
Dead sperm which take eosin stain and abnormal tail (folded tail sperm). Slide stained with Nigrosine and Eosin stain (100×).

The results presented in Table 2 and Figs. 14 and 15 indicate that the effect of treatment of Metformin, silver and silver nanoparticles, respectively, on sperm treatments, where the motility and vitality increased significantly $\left( {P \le 0.05} \right)$ for the group treated with silver nanoparticles when compared to the negative control. While the group treated with silver and Metformin, a significant $\left( {P \le 0.05} \right)$ increase in movement was observed, but with a less effect. While the abnormalities and the number of deaths were significant as a result of diabetes, where there was a decrease in mortality and Abnormalities in the treated groups.

**Table 2:** Percentage of sperms motility, Dead sperms, sperms abnormalities and the Count in treated and control groups.

Parameters (mean ± SD)	Motility (%)	Dead (%)	Abnormalities (%)	Count (× 10^7^)
Neg. control	A88.5 ± 3.11	D12.25 ± 2.22	B14.5 ± 3	A28 ± 2.94
STZ	D61.75 ± 3.59	A38.75 ± 5.19	A25.75 ± 3.86	C17.25 ± 1.708
STZ + Medformin	B80 ± 4.08	BC23 ± 3.65	AB20 ± 1.826	C19.75 ± 2.22
STZ + Ag	C72 ± 6.06	B25.5 ± 3.87	A22.25 ± 2.5	C20.5 ± 2.08
STZ + Ag-NPs	AB85 ± 4.08	C19.5 ± 2.65	B14.25 ± 6.4	B24 ± 1.826
LSD	6.484686	5.522128	5.812593	3.31266
*P*-value	0.0002	0.00005	0.003	0.00012

The results showed that the effect of silver nanoparticles on the treatments was positive and significantly increased in motility and living with a significant decrease in deformities and thus mortality in the treated groups. Silver nanoparticles prepared using the environmentally friendly garlic plant protect sperm by increasing the oxidative activity in the immune system, thus preventing reactive oxygen species from lipid peroxidation stimulation responsible for subsequent sperm damage. Our result clearly indicates the important role of silver nanoparticles in various medical applications^53,54^.

## Conclusion

In this study, silver nanoparticles were successfully generated using a simple, efficient and inexpensive protocol from fresh garlic plant as reducing agent, and silver nanoparticles with a diameter of $\left[ {50 - 350 } \right] \;{\text{nm}}$ were obtained. They were well crystallized, spherical, stable, and compatible with living cells. This study proved that silver nanoparticles have a great importance and an effective role against diabetes compared to Metformin, and silver nanoparticles succeeded in reducing the high percentage of damage in sperm transactions resulting from diabetes, and thus cause an increase in motility to reach the maximum value with reduced abnormalities.

## Data Availability

The datasets generated and/or analyzed during the current study are available in the department of biology at university of Baghdad-Iraq: biology@sc.uobaghdad.edu.iq (See attached file: “Plant identification certificate). No DNA sequence was performed in this work.
