# Author Correction: Green synthesis and characterization of silver nanoparticles for reducing the damage to sperm parameters in diabetic compared to metformin

**DOI:** 10.1038/s41598-023-33579-0

**Published:** 2023-04-24

**Authors:** Iman A. Mohammed Ali, Ali Ben Ahmed, Hazim Ismail Al-Ahmed

**Affiliations:** 1 Laboratory of Applied Physic, Faculty of Sciences of Sfax, University of Sfax, 3018 Sfax, Tunisia; 2 Ministry of Higher Education & Scientific Research’s, Baghdad, Post Office PO Box 55509, Iraq; 3 Biotechnology Research Center, University of Al-Nahrain, Baghdad, 35095 Iraq

Correction to: *Scientific Reports* 10.1038/s41598-023-29412-3, published online 08 February 2023

The original version of this Article omitted an affiliation for Iman A. Mohammed Ali. The correct affiliations are listed below.

Laboratory of Applied Physic, Faculty of Sciences of Sfax, University of Sfax, 3018 Sfax, Tunisia.

Ministry of Higher Education & Scientific Research’s, Baghdad, Post Office PO Box 55509, Iraq.

The original Article has been corrected.

